# First International Consensus Conference on lesions of uncertain malignant potential in the breast (B3 lesions)

**DOI:** 10.1007/s10549-016-3935-4

**Published:** 2016-08-13

**Authors:** Christoph J. Rageth, Elizabeth AM O’Flynn, Christopher Comstock, Claudia Kurtz, Rahel Kubik, Helmut Madjar, Domenico Lepori, Gert Kampmann, Alexander Mundinger, Astrid Baege, Thomas Decker, Stefanie Hosch, Christoph Tausch, Jean-François Delaloye, Elisabeth Morris, Zsuzsanna Varga

**Affiliations:** 1 Brust-Zentrum Zürich, Seefeldstr. 214, 8008 Zurich, Switzerland; 2 Centre du sein, Département de Gynécologie et d’Obstétrique, Hôpitaux Universitaires de Genève, Bd de la Cluse 30, 1211 Genève 14, Switzerland; 3 Institute of Cancer Research and Royal Marsden Hospital, London, UK; 4 Memorial Sloan Kettering Cancer Center, Breast and Imaging Center, 300 E 66th St Suite 723, New York, NY 10065 USA; 5 Institut für Radiologie und Nuklearmedizin, Luzerner Kantonsspital, 6000 Lucerne, Switzerland; 6 Institute of Radiology, Department of Medical Services, Kantonsspital Baden, im Ergel, 5404 Baden, Switzerland; 7 DKD HELIOS Klinik, Aukammallee 33, 65191 Wiesbaden, Germany; 8 Imagerie du Flon, Vigie 5, 1000 Lausanne 20, Switzerland; 9 Centro di Radiologia e Senologia Luganese, Corso Pestalozzi 3, 6900 Lugano, Switzerland; 10 Marienhospital Osnabrück, Bischofsstraße 1, 49074 Osnabrück, Germany; 11 Institut für Pathologie am Dietrich-Bonhoeffer-Klinikum, Salvador-Allende-Straße 30, 17036 Neubrandenburg, Germany; 12 Centre du sein, Centre Hospitalier, Universitaire Vaudois, 1011 Lausanne, Switzerland; 13 Institute of Surgical Pathology, University Hospital Zurich, Schmelzbergstrasse 12, 8091 Zurich, Switzerland

**Keywords:** B3 lesions, Vacuum-assisted biopsy, Consensus, Breast, Uncertain malignant potential, Breast surgery

## Abstract

The purpose of this study is to obtain a consensus for the therapy of B3 lesions. The first International Consensus Conference on lesions of uncertain malignant potential in the breast (B3 lesions) including atypical ductal hyperplasia (ADH), flat epithelial atypia (FEA), classical lobular neoplasia (LN), papillary lesions (PL), benign phyllodes tumors (PT), and radial scars (RS) took place in January 2016 in Zurich, Switzerland organized by the International Breast Ultrasound School and the Swiss Minimally Invasive Breast Biopsy group—a subgroup of the Swiss Society of Senology. Consensus recommendations for the management and follow-up surveillance of these B3 lesions were developed and areas of research priorities were identified. The consensus recommendation for FEA, LN, PL, and RS diagnosed on core needle biopsy or vacuum-assisted biopsy (VAB) is to therapeutically excise the lesion seen on imaging by VAB and no longer by open surgery, with follow-up surveillance imaging for 5 years. The consensus recommendation for ADH and PT is, with some exceptions, therapeutic first-line open surgical excision. Minimally invasive management of selected B3 lesions with therapeutic VAB is acceptable as an alternative to first-line surgical excision.

## Introduction

Breast lesions classified as lesions of uncertain malignant potential (B3) are a heterogeneous group of abnormalities with a borderline histological spectrum, and a variable but low risk of associated malignancy [1]. They encompass a spectrum of histological diagnoses including atypical ductal hyperplasia (ADH), flat epithelial atypia (FEA), classical lobular neoplasia (LN), papillary lesions (PL), benign phyllodes tumors (PT), and radial scars (RS), each with variable rates of upgrade or long-term increased risk of breast cancer [2]. Histological diagnosis of a B3 lesion is made by either core needle biopsy (CNB) mostly using a 14G spring-loaded CNB or by vacuum-assisted biopsy (VAB) using a 7G–11G device either under ultrasound, stereotactic, or MRI guidance following informed consent and local anesthetic. Occasionally it is an incidental finding on a specimen which has been excised surgically.

Between 4 and 9 % of all CNBs are classified as B3 lesions with numbers increasing due to advances in diagnostic imaging such as highly sensitive MRI scanning and interventional techniques such as VAB [3]. However, the positive predictive value for malignancy has been falling (from 29 to 10 %) [4, 5]. Management of B3 lesions provides a challenge to the multidisciplinary team as diagnostic surgical excision is no longer the only available treatment. Minimally invasive breast biopsy, or VAB, facilitates removal of larger volumes of tissue than a CNB equivalent to a small wide local excision and allows the same diagnostic accuracy as open surgery [6]. For many B3 lesions, instead of surgical excision, VAB may be sufficient for therapeutic excision which would benefit the patient and save on healthcare costs by obviating the need for surgery [7].

The evidence base for appropriate management of B3 lesions in the breast is limited. Practice varies greatly from country to country. This article provides a review of the literature for the six different B3 lesions documented including the analysis of 2 large Swiss B3 histology databases followed by consensus recommendations by an expert panel taken after a voting by the participants of the symposium organized by the International Breast Ultrasound School (IBUS) and the Swiss MIBB group—a subgroup of the Swiss Society of Senology in January 2016, in Zurich, Switzerland.

## Methodology

The first International Consensus Conference on lesions of uncertain malignant potential (B3) was held with international experts as part of the IBUS seminar in January 2016 in Zurich. These meetings have been held bi-annually since 2001 with discussion of therapeutic management options for B3 lesions. The meeting in January 2016 had 90 participants with the multidisciplinary expert panel (all the aforementioned authors) comprising nine radiologists, two pathologists, one surgeon, and three gynecologists. Each of the B3 lesions was discussed in turn with reference to the published literature and the analysis of the MIBB [8] working group database (histology from 22,072 VABs).

A set of recommendations for the management of B3 breast lesions was prepared building on the current practice of the Swiss MIBB working group. Recommendations for management of B3 breast lesions following histological diagnosis were either: (i) surveillance (defined as 6 monthly or yearly mammography and/or ultrasound, depending on the imaging findings) [9], (ii) therapeutic VAB excision, or (iii) therapeutic open surgical excision. All participants at the Consensus Conference were invited to vote on all recommendations and 50 of the 90 participants decided to. 27 (57 %) were radiologists, 2 (4 %) pathologists, 2 (4 %) surgeons, and 16 (34 %) gynecologists. Nearly two-thirds of those voting had more than 10 years’ experience in breast disease diagnosis and management.

There were 3344 “pure” B3 lesions in the MIBB database (15 % of all lesions). Following presentations of each B3 lesion in detail reviewing the published literature, three questions were asked in turn:

Q1. If a CNB returned a B3 lesion on histology, should the lesion be therapeutically excised?

Q2. If so, should it be excised therapeutically using VAB?

Q3. If the VAB returned a B3 lesion on histology and if the lesion was completely removed on imaging, is surveillance acceptable or should a repeat VAB or surgical excision be performed?

A panel discussion followed the voting and consensus recommendations were agreed for the management of each B3 lesion.

## Results

Table 1 illustrates the number of cases in each B3 lesion category that underwent therapeutic surgical excision compared to those that did not following VAB. Table 2 illustrates the upgrade rate to invasive malignancy for each B3 lesion in cases that underwent therapeutic open surgical excision following VAB. Table 3 documents the voting results for each of the B3 lesions and Table 4 shows the overall consensus recommendations for the management of B3 lesions.

**Table 1:** MIBB (VAB only cases) database records indicating numbers of the different B3 lesions that underwent therapeutic surgical excision following VAB and those that did not

	*N*	Number of cases without therapeutic open surgical excision	Number of cases with therapeutic open surgical excision	unknown
Atypical ductal hyperplasia	736	239 (33 %)	439 (60 %)	58 (8 %)
Flat epithelial atypia	773	521 (67 %)	177 (23 %)	75 (10 %)
Classical lobular neoplasia	546	313 (57 %)	191 (35 %)	42 (8 %)
Papillary lesion	954	683 (72 %)	154 (16 %)	117 (12 %)
Benign phyllodes tumor	18	13 (72 %)	3 (17 %)	2 (11 %)
Radial scar	317	235 (74 %)	46 (15 %)	36 (11 %)
Total	3344	2004	1010	330

**Table 2:** Illustrates the upgrade rate to invasive malignancy for each B3 lesion in cases that underwent therapeutic open surgical excision following VAB

	Number of cases with therapeutic open surgical excision	Upgrade rate	Numbers upgraded to DCIS (B5a)	Numbers upgraded to invasive malignancy (B5b)
Atypical ductal hyperplasia	439	121 (27.6 %)	99 (22.6 %)	22 (5.0 %)
Flat epithelial atypia	177	35 (19.8 %)	19 (10.7 %)	16 (9.0 %)
Classic lobular neoplasia	191	48 (25.1 %)	24 (12.6 %)	24 (12.6 %)
Papillary lesion	154	12 (7.8 %)	8 (5.2 %)	4 (2.6 %)
Phyllodes tumor	3	0 (0 %)	0 (0 %)	0 (0 %)
Radial scar	46	5 (10.9 %)	4 (8.7 %)	1 (2.2 %)
Total	1010	221 (21.9 %)	155 (15.3 %)	67 (6.6 %)

**Table 3:** Illustrates the voting results for each of the B3 lesions

	If a histological diagnosis of a B3 lesion is made on CNB			If the B3 lesion should be excised			If a B3 lesion has been therapeutically excised on VAB			
	The lesion should be excised?	The lesion should not be excised?	No vote	Therapeutic VAB is acceptable?	Therapeutic open surgical excision should be performed?	No vote	An open re-excision should be performed?	A repeat VAB should be performed?	Surveillance is acceptable?	No vote
ADH	46 (100 %)	0 (0 %)	0 (0 %)	11 (24 %)	33 (73 %)	1 (2 %)	23 (51 %)	1 (2 %)	19 (42 %)	2 (4.4 %)
FEA	36 (97 %)	0 (0 %)	1 (3 %)	26 (70 %)	10 (27 %)	1 (3 %)	1 (3 %)	1 (3 %)	36 (94 %)	0 (0 %)
LN	32 (91 %)	1 (3 %)	2 (6 %)	19 (58 %)	14 (42 %)	0 (0 %)	5 (13 %)	0 (0 %)	33 (87 %)	0 (0 %)
PL	40 (100 %)	0 (0 %)	0 (0 %)	32 (84 %)	4 (11 %)	2 (5 %)	4 (9 %)	0 (0 %)	39 (91 %)	0 (0 %)
PT	32 (91 %)	1 (3 %)	2 (6 %)	19 (51 %)	17 (46 %)	1 (3 %)	5 (11 %)	1 (2 %)	34 (83 %)	1 (2 %)
RS	41 (85 %)	4 (8 %)	3 (6 %)	33 (72 %)	12 (26 %)	1 (2 %)	1 (2 %)	0 (0 %)	47 (98 %)	0 (0 %)

**Table 4:** Consensus recommendations for the management of B3 lesions. *FEA* flat epithelial atypia, *RS* radial scar, *PL* papillary lesion, *PT* phyllodes tumor, *LN* classical lobular neoplasia, *ADH* atypical ductal hyperplasia, *VAB* Vacuum assisted biopsy, *OE* Open excision

	Diagnosis made by CNB	Diagnosis made by VAB
ADH	OE. VAB in unifocal ADH in small lesions could be justified	OE. If the lesion has been removed completely and only focal ADH with calcifications exists, surveillance could be justified
FEA	VAB or OE of visible lesion	surveillance is justified if the radiological lesion has been removed
LN^a^	OE or VAB (remove US-visible lesion)	OE. High risk follow-up if the radiological lesion has been removed
PL^b^	Remove larger or symptomatic (and especially peripheral) Papillomas. VAB is Acceptable	
PT	OE. Free margins in borderline and malignant PT’s	Follow up in completely excised benign PT’s surveillance is justified
RS	VAB or OE of visible lesion	surveillance is justified if the radiological lesion has been removed

^a^ *LN* only classical type. Pleomorphic LN should not be classified as B3 lesion. It is rather being treated like a high grade DCIS

^b^PL with atypia: Such a lesion should not be classified as papilloma, but rather as FEA or ADH according to the type of atypia found

### Atypical ductal hyperplasia

The histopathologic features of ADH are essentially those of low-grade ductal carcinoma in situ (DCIS). If less than 2 mm, the lesion is classified as ADH and if more than 2 mm, it is classified as low-grade DCIS [10, 11]. This is the fundamental problem of ADH diagnosed by CNB where often only parts of the lesion have been excised as VAB or surgical excision may upgrade the diagnosis from a B3 to a B5a lesion and this is why most guidelines recommend surgical excision following a CNB diagnosis of ADH [12]. Stereotactic VAB underestimation rates range from 9 to 58 % [13–25]. Even with complete removal of malignant microcalcifications by VAB, underestimation rates up to 17 % are documented [26–29]. The highest underestimation rates (22–65 %) are published for ultrasound-guided 14G CNB [21, 22, 25, 30–34] while ultrasound-guided VABs have much lower rates of underestimation (0–22 %) [33, 35]. Grady et al. found no underestimation in lesions completely removed by 8 G ultrasound-guided VAB [35]. For MRI-guided VAB only two studies exist regarding underestimation of ADH showing underestimation rates of 32 and 38 %, respectively [36, 37].

In studies analyzing patients on surveillance without surgical treatment following a VAB diagnosis of ADH, long-term upgrade rates to invasive breast cancer of 3–8 % are reported [19, 26]. After therapeutic surgical excision of ADH, patients had a fourfold increased risk of developing breast cancer in either breast with a cumulative incidence of 30 % in 25 years [10, 11, 38, 39]. Currently, there is very little data to indicate that lesions smaller than 6 mm completely excised by VAB with less than 2 foci of ADH may safely avoid surgery [14, 19, 21, 26, 40].

736 cases of ADH from the MIBB database were reviewed. 439 (60 %) had subsequent therapeutic open surgical excision following VAB (Table 1) with an upgrade rate of 5 % (22/439) to invasive malignancy (B5b) (Table 2).

### If a CNB returned ADH on histology

100 % of the participants thought the lesion should be excised. 24 % thought therapeutic VAB excision was acceptable and 73 % thought therapeutic open surgical excision should be performed.

### If a VAB returned ADH on histology

51 % of the participants thought that therapeutic open surgical excision should be performed and 42 % thought that surveillance was adequate (Table 3).

#### Consensus recommendation

A lesion containing ADH which is visible on imaging should undergo therapeutic open surgical excision. If a unifocal ADH lesion^1^ has been completely removed by VAB, surveillance is justified. Otherwise open surgery is still recommended (Table 4).

### Flat epithelial atypia

FEA is defined as a neoplastic proliferation of the terminal ductulo-lobular units (TDLU) by a few layers of cells with low-grade (monomorphic) atypia [2, 41, 42]. Histopathology of FEA lesions encompasses the proliferation of round and uniform cells (defined as low-grade atypia) exhibiting inconspicuous nuclei [2, 41, 42]. There is often associated calcification. An FEA lesion lacks secondary architecture such as roman bridges or cellular tufts and exhibits a characteristic immunophenotype of negative low-molecular weight cytokeratins and high regulation of estrogen receptors [2, 41, 42]. The mammographic appearance of FEA is mostly seen as microcalcifications which are irregular and branching with accompanying marked duct dilatation [2, 41, 42]. Coexisting lesions both on imaging and on histopathology encompass classical LN, other benign columnar cell lesions, low-grade intraductal proliferations such as ADH/DCIS, or tubular carcinoma [2, 41, 42].

The risk of developing breast cancer with a diagnosis of FEA is estimated at 1–2 times higher than those without FEA [2, 41, 42]. ADH and DCIS are the most frequent pathologies found following surgical excision with their incidence varying from 0 to 40 %. Underestimation rates are between 0–20 % if FEA is diagnosed on core biopsy and are very similar if diagnosed on VAB (0–21 %) [43–48]. Current German (AGO) 2015 guidelines [12], do not recommend therapeutic open surgical excision of FEA diagnosed on CNB or VAB if the lesion is small (maximum 2 TDLU) and the imaging abnormality was completely removed by VAB. Surgical excision is recommended if there is radiopathological discrepancy, if the lesion is visible on imaging and the imaging classification is BIRADS 4. For BIRADS 3 lesions, completely removed by VAB, open surgery is not considered necessary [43–48].

773 cases of FEA from the MIBB database were reviewed. 177 (23 %) had subsequent therapeutic open surgical excision following VAB (Table 1) with an upgrade rate of 9 % (16/191) to invasive malignancy (B5b) (Table 2).

### If a CNB returned FEA on histology

97 % of the participants thought the lesion should be excised. 70 % thought therapeutic VAB excision was acceptable and 27 % thought therapeutic open surgical excision should be performed.

### If a VAB returned FEA on histology

3 % of the participants thought that therapeutic open surgical excision should be performed and 94 % thought that surveillance was adequate (Table 3).

#### Consensus recommendation

A lesion containing FEA, which is visible on imaging should undergo therapeutic excision with VAB. Thereafter surveillance is justified (Table 4).

### Classical lobular neoplasia

Classical LN encompasses a spectrum of atypical epithelial proliferations in the TDLU of the breast [2, 41, 42]. The histology consists of non-cohesive proliferating epithelial cells with or without pagetoid involvement of the terminal ducts [2, 41, 42]. There are several nomenclatures used for LN: The classical type of LN covers all lobular lesions, which develop in the TDLU except those with pleomorphic or extensive variants. The older nomenclature of atypical lobular hyperplasia (ALH) and lobular carcinoma in situ (LCIS) refers to the same lesion but to different extents, defined as ALH if less than 50 % of the given TDLU are involved and LCIS if more than 50 % is involved [2, 41, 42]. The World Health Organization (WHO) also applies the term lobular intraepithelial lesion (LIN), which can be classified as LIN 1, 2, and 3, with LIN 1 formally being equivalent to ALH, LIN2 to LCIS, and LIN3 to the pleomorphic or extensive LN variants with or without necrosis [2, 41, 42]. MIBB classification of lobular neoplasia categorizes all lesions (classical LN, ALH, LCIS, LIN1, LIN2) as B3, but LIN 3 or pleomorphic LN or those with extensive necrosis are classified as B5a. The MIBB classification and the WHO recommend the use of the histological terms classical LN as B3 and pleomorphic LN as B5a [2, 12].

The incidence of classical LN has been increasing and varies from 0.5 to 4 %. It can occur at all ages but predominantly in premenopausal women. Most lesions present incidentally without any palpable mass and less than half of classical LN lesions have associated calcification. Published data on the risk of developing breast cancer after diagnosis on CNB or VAB show, a relative risk of 1–2 % per year, 15–17 % after 15 years, and 35 % after 35 years with relatively equal rates of ipsi- and contralateral breast cancer (8.7 and 6.7 %, respectively) [2, 49–53]. The upgrade rate after classical LN diagnosis on CB or VAB is variable in the literature, ranging from 0 to 50 % which can at least partially be explained by variation in study design and inconsistent use of ALH, LCIS, and LN nomenclatures [2, 41]. In one study, underestimation was found to be 4 % in classical LN cases when LN was an incidental finding and 18 %, when LN represented the radiologic targets by D’Alfonso et al. [50]. Higher upgrade rates were associated with cases that demonstrated mass lesions and calcification on imaging or with radiopathological discordance. Lower underestimation rates were detected in classical LN cases, where no residual calcification was found after biopsy, calcification was incidental, and there was complete concordance between histological and imaging findings [2, 41, 50].

The WHO recommends surgical excision after classical LN diagnosis on CNB or VAB if there is another B3 lesion present, if another coexisting lesion warrants excision alone, if there is a mass lesion on imaging, or in any case of radiopathological discordance [2, 41]. The German AGO 2015 guidelines favor open excision only if there is a B5a component, if classical LN is extensive, in the presence of necrosis on the CNB or VAB, or in cases of discordance with imaging [12]. Open surgical excision is therefore not considered necessary if there is a complete concordance between histology and imaging, if the imaging finding is classified as BIRADS 3, or of LN is a focal finding and is not associated with calcifications [2, 12, 41].

546 cases of classical LN from the MIBB database were reviewed. 191 (35 %) had subsequent therapeutic open surgical excision following VAB (Table 1) with an upgrade rate of 12.6 % (24/191) to invasive malignancy (B5b) (Table 2).

### If a CNB returned classical LN on histology

91 % of the participants thought the lesion should be excised. 58 % thought therapeutic VAB excision was acceptable and 42 % thought therapeutic open surgical excision should be performed.

### If a VAB returned classical LN on histology

13 % of the participants thought that therapeutic open surgical excision should be performed and 87 % thought that surveillance was adequate (Table 3).

#### Consensus recommendation

A lesion containing classical LN lesion, which is visible on imaging should undergo therapeutic excision with VAB. Thereafter surveillance is justified (Table 4).

### Papillary lesion

PLs represent up to 5 % of all biopsied breast lesions [54–57]. The term PL comprises a heterogeneous group of epithelial lesions such as intraductal papilloma, intraductal papilloma with ADH, intraductal papilloma with DCIS, papillary DCIS, encapsulated papillary carcinoma, solid papillary carcinoma, and invasive papillary carcinoma [2]. PLs demonstrate intra-lesional heterogeneity and can be associated with small foci of ADH or DCIS within the PL or in the adjacent tissue which may be missed by limited sampling with CNB. When describing PL, only PL without atypia should be considered, as a lesion with atypia should be considered within the higher class lesions (e.g., ADH) and offered therapeutic open surgical excision.

Upgrade rates after surgical excision of benign papillomata diagnosed following CNB or VAB vary from 0 to 28 % with atypical cells and from 0 to 20 % for invasive cancer [58–60]. Generally, understaging of invasive malignancy is reduced if multiple biopsy cores are taken or if a larger biopsy needles are employed such as in VAB. Only one study by Chang et al. evaluated the accuracy of VAB in PL without atypia by open surgical excision following VAB with no upgrade to malignancy but an upgrade of 18.3 % to atypia [61]. Most studies following up VAB excision of PL without atypia did not observe any upgrade to malignancy with at least 2 years of surveillance [60, 62, 63]. One recorded a minimal underestimation of 1.4 % [64] and another 3.2 % [65].

The upgrade rate to malignancy following VAB in the MIBB database was 7.7 % for PL without atypia which is higher than in the documented literature. One reason might be the fact that the size of the PL was not recorded, implying that some PL might not have been completely removed. Mosier et al. removed only lesions smaller than 15 mm (range 3–15 mm) to ensure the complete removal of the PL [62]. With this approach, they had no upgrades to malignancy after nearly 9 years. Due to difficulties in excluding malignancy with small tissue samples at CNB, heterogeneity of PLs, and an upgrade rate to carcinoma of up to 20 % [58], the current recommendation is to completely remove PL without atypia, either by surgery or VAB [58–60].

954 cases of PL from the MIBB database have been reviewed. 154 (16 %) had subsequent therapeutic open surgical excision following VAB (Table 1) with an upgrade rate of 2.6 % (4/154) to invasive malignancy (B5b) (Table 2).

### If a CNB returned PL on histology

100 % of the participants thought the lesion should be excised. 84 % thought therapeutic VAB excision was acceptable and 11 % thought therapeutic open surgical excision should be performed.

### If a VAB returned PL on histology

9 % of the participants thought that therapeutic open surgical excision should be performed and 91 % thought that surveillance was adequate (Table 3).

#### Consensus recommendation

A PL lesion, which is visible on imaging should undergo therapeutic excision with VAB. Thereafter surveillance is justified (Table 4).

### Phyllodes tumor

PTs are rare fibroepithelial neoplasms accounting for less than 1 % of primary breast tumors [2, 66]. Histologically, they are classified as benign, borderline, and malignant with the first two subtypes categorized as B3 lesions [67]. The majority of PTs are benign, (63–78 %) with borderline PTs diagnosed in 11–30 % of cases [2]. Incidence is highest in women aged 40–51 years [68, 69]. Overlapping clinical, radiological, and histopathological features may make differentiation from benign fibroadenomata challenging at times, however accurate preoperative diagnosis is essential to establish the most appropriate therapeutic approach.

Underestimation rates of PTs following CNB range from 8 to 39 % (mean 20 %) [70, 71].Concordance rates between CNB and surgical excision for benign and borderline/malignant PTs are between 38.5 and 82 % and 74.7–100 %, respectively [72], with higher concordance of up to 90 % following VAB [73]. Youk et al. documented upgrades from benign to malignant PTs in 8.7 % of patients, with higher underestimation rates found in pre-excisional ultrasound BIRADS 4 lesions and higher classifications [73]. Recurrence rates for benign PTs are similar following ultrasound -guided VAB (0–19.4 %) and surgical excision (5–17 %) [74–76], but higher for borderline PTs following surgical excision (14–25 %) [77, 78]. The majority of published studies recommend open surgical excision for all histological PT-subtypes, despite the fact that the recurrence rate for benign PTs after VAB and surgical excision do not vary significantly [73–76, 79–81].

18 cases of PT from the MIBB database have been reviewed. 3 (17 %) had subsequent therapeutic open surgical excision following VAB (Table 1) with an upgrade rate of 0 % to invasive malignancy (B5b) (Table 2).

### If a CNB returned PT on histology

91 % of the participants thought the lesion should be excised. 51 % thought therapeutic VAB excision was acceptable and 46 % thought therapeutic open surgical excision should be performed.

### If a VAB returned PT on histology

11 % of the participants thought that therapeutic open surgical excision should be performed and 83 % thought that surveillance was adequate (Table 3).

#### Consensus recommendation

A PT lesion, which is visible on imaging should undergo therapeutic open surgical excision with clear margins. If a VAB shows a benign PT, surveillance is justified, while borderline and malignant PTs require re-excision to obtain clear margins (Table 4).

### Radial scar

RS or complex sclerosing lesions (CSL) of the breast are characterized by a stellate-like distortion. The nomenclature depends on the size of the lesion which is defined as radial scar if the focus is less than 1 cm or complex sclerosing lesion if over 1 cm [2, 41]. Histopathology of a RS/CSL involves a stellate-like elastosis with or without the presence of associated lobulocentric cysts, usual ductal hyperplasia, adenosis, and microcalcifications. The adenosis may evolve the elastic fibers resulting in entrapped glands, which may mimic a highly differentiated neoplastic glandular proliferation [2, 41]. On mammography, RS/CSL mostly appear as a stellate lesion which mimics an invasive carcinoma. The incidence is variable being 4–9 % in population-based pathology databases, but being significantly higher, up to 63 % in sole pathology literature [2, 41].

The prognosis of RS/CSL depends on the presence of associated atypia [2, 41, 82–87]. Based on correlation between imaging and pathology, RS/CSL without atypia following CNS or VAB are unlikely to have malignancy in the surgical excision specimen if the lesion is less than 6 mm on imaging and the patients are younger than 40 years or older than 60 years [82–87]. The relative risk of developing breast cancer given the presence of a RS/CSL without atypia varies between 1.1 and 3.0 % [88–90]. Conversely, RS/CSL showing cytological or histological atypia, have a higher relative risk of 2.8–6.7 % particularly in patients over 50 years of age [88–90]. Underestimation rates for pure RS/CSL vary between 1 and 28 % following CNB and 8 % following VAB [2, 41, 82–87]. The AGO 2015 and WHO 2012 guidelines recommend surveillance if the imaging findings have been completely excised at VAB and no atypia was found in the histological examination. RS/CSL with atypia on histology following CNB/VAB should undergo therapeutic open surgical excision [2, 12, 41, 88–90].

317 cases of RS/CSL from the MIBB database have been reviewed. 46 (15 %) had subsequent therapeutic open surgical excision following VAB (Table 1) with an upgrade rate of 2 % (1/46) to invasive malignancy (B5b) (Table 2).

### If a CNB returned RS/CSL on histology

85 % of the participants thought the lesion should be excised. 72 % thought therapeutic VAB excision was acceptable and 26 % thought therapeutic open surgical excision should be performed.

### If a VAB returned RS/CSL on histology

2 % of the participants thought that therapeutic open surgical excision should be performed and 98 % thought that surveillance was adequate (Table 3).

#### Consensus recommendation

A RS/CSL lesion, which is visible on imaging should undergo therapeutic excision with VAB. Thereafter surveillance is justified (Table 4).

## Discussion

The expert consensus panel and participants agreed that for most of the B3 lesions (except for ADH and PT), surgery can be avoided and therapeutic excision with VAB of a lesion which is visible on imaging is an acceptable alternative. However, as data are lacking at present the panel still recommends open surgery in cases of ADH. As more data on the minimally invasive conservative management of B3 lesions become available, a more conservative approach may also be justified in cases of ADH. The outcome from this consensus meeting is a progressive move forward to a more conservative approach to managing these lesions in which open surgery can potentially be avoided. Studies following a diagnosis of low-grade DCIS have shown excellent survival rates of more than 98 % at ten years after diagnosis without surgery [91, 92] which have prompted randomized phase III trials for surgery versus no surgery in low- and intermediate-grade DCIS [93, 94]. Therefore it is becoming clear, that it is even more reasonable to try to avoid unnecessary open surgery or overtreatment in some women. It is important to emphasize that these recommendations cannot exclude a false-negative diagnosis in every individual patient and each case should be discussed on an individual basis with a multidisciplinary team taking into account the imaging features, lesion size, practicality and technical feasibility of minimally invasive management, patient demographics, and patient preference.

## Footnotes

1 Focal ADH is not defined in the WHO classification and mentioning the exact dimension of ADH lesions below 2 mm is not mandatory. However recent literature data suggest, that ADH lesions (less than 2 mm in extension max. 2 cross sections) may not have to undergo surgical excision. These data need further validation.
